# Bovine besnoitiosis emerging in Central-Eastern Europe, Hungary

**DOI:** 10.1186/1756-3305-7-20

**Published:** 2014-01-13

**Authors:** Sándor Hornok, András Fedák, Ferenc Baska, Regina Hofmann-Lehmann, Walter Basso

**Affiliations:** 1 Department of Parasitology and Zoology, Faculty of Veterinary Science, Szent István University, Budapest, Hungary; 2 Veterinary Authority, Miskolc, Hungary; 3 Department of Pathology, Faculty of Veterinary Science, Szent István University, Budapest, Hungary; 4 Clinical Laboratory, Vetsuisse Faculty, University of Zurich, Zurich, Switzerland; 5 Institute of Parasitology, Vetsuisse Faculty, University of Zurich, Zurich, Switzerland; 6 Department of Farm Animals, Vetsuisse Faculty, University of Zurich, Zurich, Switzerland

**Keywords:** *Besnoitia besnoiti*, Cattle, Mechanical vector, Vector-borne, Emerging disease

## Abstract

**Background:**

*Besnoitia besnoiti*, the cause of bovine besnoitiosis, is a cyst-forming coccidian parasite that has recently been shown to be spreading in several Western and Southern European countries.

**Findings:**

Clinical cases of bovine besnoitiosis were confirmed for the first time in Hungary, by histological, serological and PCR analyses.

**Conclusions:**

This is the first report of autochthonous bovine besnoitiosis in Central-Eastern Europe. The emergence of bovine besnoitiosis in this region represents a further example, when human activity (i.e. cattle trading) is the main factor involved in the geographical spread of an infectious disease.

## Findings

### Background

*Besnoitia besnoiti* is a protozoan parasite belonging to the group of cyst-forming coccidia (Apicomplexa, Sarcocystidae). It has been assumed that *B. besnoiti*, similarly to other *Besnoitia* species, has a heteroxeneous life cycle with intermediate and final host. Cattle is the most important intermediate host species of *B. besnoiti*, while its final host remains unknown [1]. During acute bovine besnoitiosis multiplication of the parasite takes place in vascular endothelium with clinical manifestations including mainly oedema of the head, neck, and later of the legs (anasarca stage), as well as respiratory signs [2]. The chronic (scleroderma) stage of the infection (so-called elephant skin disease) is a severe, debilitating condition with skin thickening, wrinkling and hair loss attributable to large numbers of cysts in the skin and subsequent host inflammatory reaction around approx. 30% of tissue cysts [3]. Infection of testes frequently entails infertility of bulls, and cows may abort [2]. Although mortality is usually low, morbidity, decreased production and culling of infected cattle may lead to significant economical losses.

Mechanical transmission by blood-sucking arthropods (most notably by tabanid horse flies and the stable fly, *Stomoxys calcitrans*) and iatrogenically, through repeated usage of hypodermic needles are the only experimentally proved routes of transmission [4,5]. However, there is epidemiological evidence in support of other modes of infection, i.e. close contact between animals [2]. The presence of cysts in the genital mucosae may probably result in transmission during mating. In addition, recovered and subclinically infected animals are thought to remain life-long carriers of *B. besnoiti*, and are considered as important sources of infection for naïve cattle [2,6,7]. Some wild ruminant species also appear to be susceptible to *B. besnoiti* infection [2,8].

**Figure 1:**
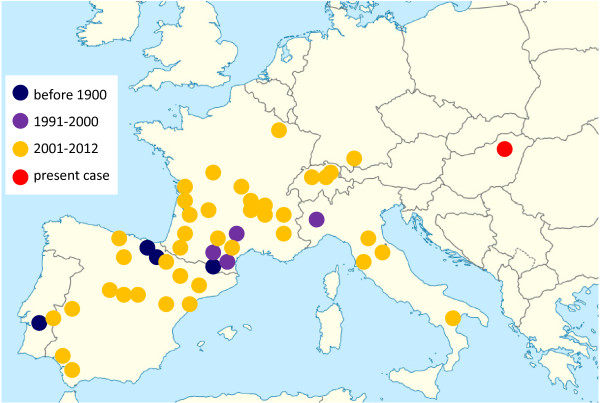
**Map of Europe showing the spread of bovine besnoitiosis in chronological order, updated from **[2,7]**and including the geographical location of the present case.**

In Europe bovine besnoitiosis was a neglected disease, restricted to endemic foci in the Pyrenees (Southern France) and Alentejo region (Portugal) [2] (Figure 1). During the last decade, a significant geographical expansion occurred towards other parts of the endemic countries (Spain and France), as well as to other countries considered free of bovine besnoitiosis, such as Germany, Italy and Switzerland [2,7]. Most vector-borne infections emerging in new regions of Europe show a northward spread, associated with northern establishment of their biological vectors due to climate change. Unlike these, *B. besnoiti* has suitable and indigenous mechanical vectors on most parts of our continent, and therefore exhibited eastward emergence mainly related to the introduction of cattle imported from France [2,7]. Accordingly, progressive establishment of bovine besnoitiosis was hitherto observed in countries neighbouring France (Figure 1).

### Findings and discussion

A beef cattle farm located in Eastern Hungary (Figure 1), with a herd of 670 animals (including eight bulls) at the beginning of the study, had imported a total of 178 apparently healthy Aubrac heifers from France in 2011, followed by a further import of three Aubrac bulls during the spring of 2012 for breeding purposes. Cattle were kept extensively, grazing pastures from May till November. Bulls, when not serving cows, were kept stabled. Calves remained with their mothers until weaning at six to seven months of age. Clinical manifestations resembling besnoitiosis, i.e. leg oedema and skin thickening was first observed in one of the imported Aubrac heifers in May 2012. During late winter in 2013 malformed newborn calves, (with compressed skull and short legs) were delivered by three Aubrac cows. Routine virological and bacteriological evaluation did not reveal the presence of any fetopathogenic agents (BVD or IBR virus, *Leptospira*, *Brucella*, *Chlamydia*, *Coxiella*) that are known to occur in the region. However, because evaluation of tissues in malformed calves for the presence of *B. besnoiti* was not possible at that time, and only abortions (but not foetal malformations) were reported in association with bovine besnoitiosis [2], these pathologies may not necessarily have been a consequence of *B. besnoiti* infection.

**Figure 2:**
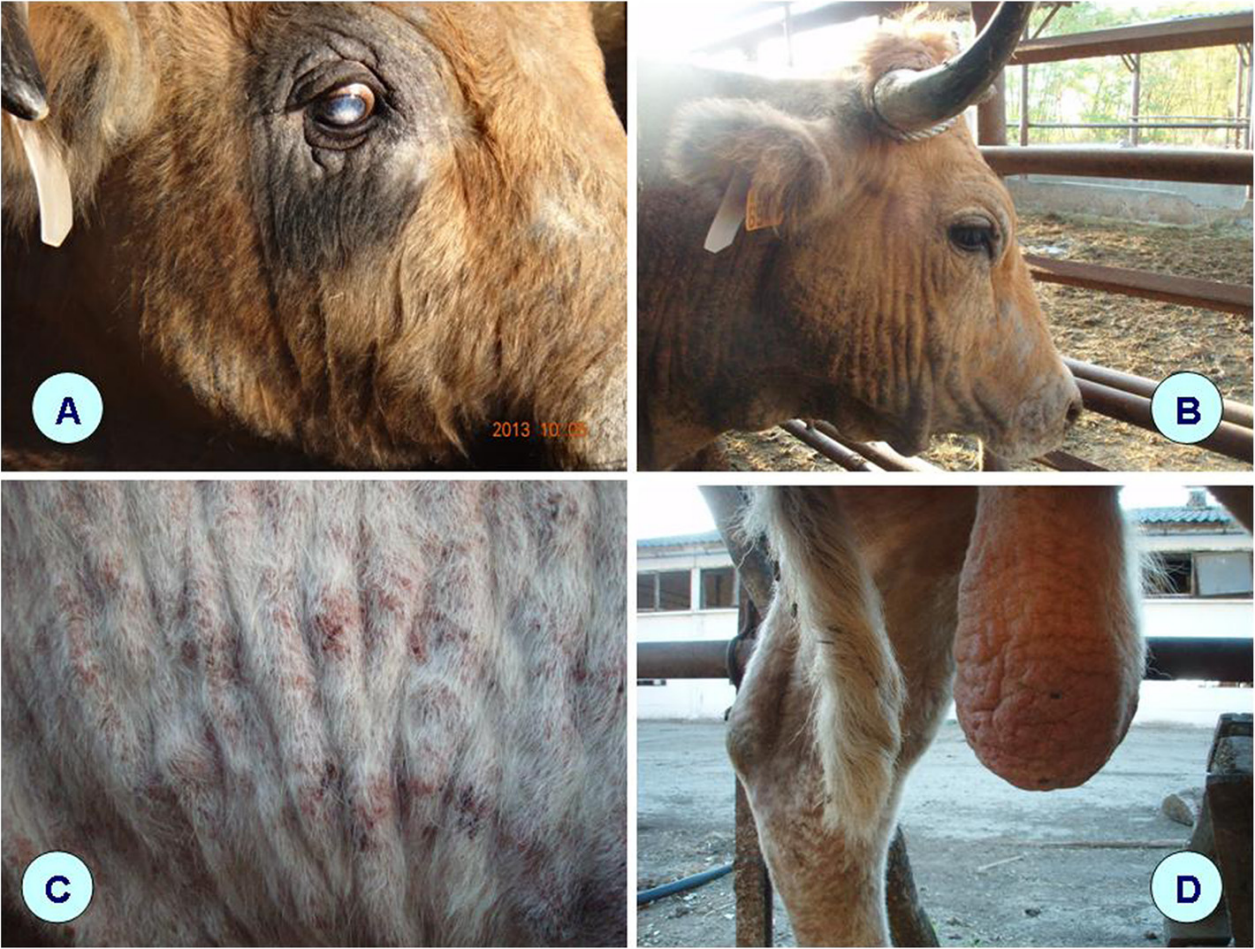
Clinical signs of besnoitiosis: (A) periorbital lichenification in an Aubrac bull; (B) alopecia and wrinkling of the skin on the head and neck of an Aubrac cow; (C) sides of a local Charolais cow showing similar lesions; (D) chronic orchitis in a Charolais bull.

Since May 2013 the veterinarian in charge of the farm reported more animals with suspected besnoitiosis, including both imported (Aubrac) and local (Charolais) cattle (Table 1). The first clinical sign noted in all affected Aubrac cows and in one bull was oedema on the lower parts of the legs, followed by gradually aggravating skin lesions during the summer months, such as wrinkling and alopecia on the neck and head region, lateral and dorsal aspects of the trunk, together with orchitis in the bull (Figure 2). Three Aubrac calves became diseased in August (2013) on the pasture, showing dyspnoea and nasal discharge (without skin lesions). At the same time two adult, locally born Charolais cattle, which were stabled, also developed clinical signs, i.e. the cow exhibited respiratory signs, and the bull developed oedema of the legs, severe skin lesions and orchitis (Table 1).

**Table 1:** **Data of sampled cattle and results of histological, molecular and serological analyses for the detection of *B. besnoiti *** **infection**

**Breed**	**Sex (age in years)**	**History**	**Clinical signs**	**Presence of tissue cysts**	**PCR (Ct)**	**ELISA (PP)**	**IFAT (titre)**	**WB**
	Bull (2.5)	Locally born	Leg oedema, skin lesions, orchitis	+	+ (17.7)	+ (66.17)	3200	+
**Charolais**	Cow (3)	Locally born	Leg oedema, skin lesions	+	+ (16.7)	+ (113.95)	3200	+
	Cow (6)	Locally born	Respiratory signs	ne.	+ (18.4)	+ (96.37)	3200 ≤	+
	Bull (4)	Imported	Leg oedema, skin lesions, orchitis	+	+ (24.9)	+ (78.08)	1600	+
	Cow (3.5)	Imported	Leg oedema, skin lesions	+	+ (17)	+ (107.95)	3200 ≤	+
	Cow (3.5)	Imported	Leg oedema, skin lesions	ne.	+ (18.4)	+ (114.84)	3200 ≤	+
	Cow (3.5)	Imported	Leg oedema, skin lesions	ne.	+ (17.6)	+ (106.54)	3200 ≤	+
**Aubrac**	Calf (0.5)	Locally born	Nasal discharge, dyspnoea	ne.	nd.	+ (90.83)	1600	+
	Calf (0.5)	Locally born	Nasal discharge, dyspnoea	ne.	nd.	+ (81.97)	800	+
	Calf (0.5)	Locally born	Nasal discharge, dyspnoea	ne.	nd.	+ (95.62)	3200	+
	Bull (3.5)	Imported	None	ne.	nd.	+ (92.90)	800	+
	Bull (3.5)	Imported	None	ne.	nd.	+ (72.18)	800	+

Abbreviations: ne. - not evaluated, nd. - not done.

In the autumn of 2013 blood samples were drawn by coccygeal venipuncture from all ten animals that had shown signs resembling besnoitiosis, as well as from two Aubrac bulls without clinical signs, for serological analysis (Table 1). The rest of the herd was apparently normal. In the case of seven out of these twelve animals skin samples were obtained from the neck region with a Biopsy Punch (Stiefel Laboratories Ltd., Sligo, Ireland) after local anaesthesia. In four animals the skin sample was divided into three parts: the first two parts were fixed with 8% formalin or Bouin solution, respectively, and processed for histopathology, while the third part was prepared for PCR analysis together with skin samples of a further three animals. DNA was extracted from the latter samples with QIAamp DNA Mini Kit (QIAGEN, Hilden, Germany) according to the manufacturer’s instructions, and a real-time PCR based on sequence amplification of the internal transcribed spacer region 1 (ITS-1) of the ribosomal RNA gene of *B. besnoiti* was performed [9]. Serum samples were evaluated with three serological methods having different sensitivities and specificities: a commercial ELISA (PrioCHECK Besnoitia Ab 2.0, Prionics, Zurich, Switzerland), IFAT and WB using *B. besnoiti* tachyzoite antigen as previously described [7]. The criteria of positivity were: in the commercial ELISA, a sample extinction value expressed as the percentage of that of the positive control (PP value) of 20 or above according to the manufacturers’ instructions; IFAT titre of at least 100, and recognition of at least four out of ten selected tachyzoite antigens in Western blot. Results of sample analyses are summarized in Table 1.

**Figure 3:**
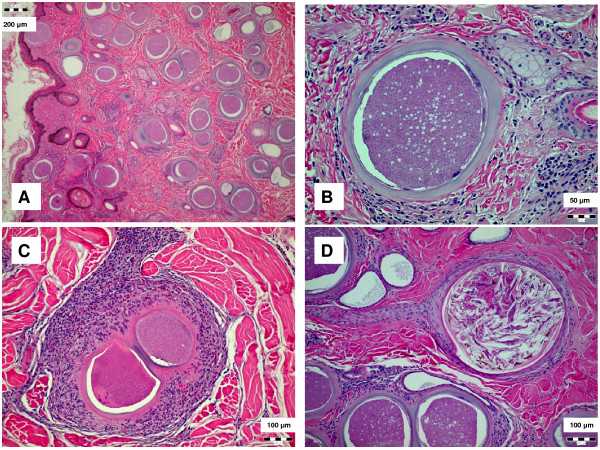
Histopathological changes during besnoitiosis: (A) numerous tissue cysts in the dermis and epithelial desquamation; (B) cyst showing three-layered wall: outermost connective tissue, middle layer containing host cell nuclei and the internal parasitophorous vacuole membrane surrounding the bradyzoites; (C) infiltration with histiocytes and eosinophilic granulocytes around cysts; and (D) occlusion of sebaceous gland duct.

Antibodies against *B. besnoiti* were detected in all twelve animals, including the two asymptomatic Aubrac bulls, by the three serological methods used. The infection was further confirmed by histopathology and real-time PCR in all analyzed skin biopsy samples from cattle with clinical signs (Table 1). Histology revealed multiple, large cysts (100–400 μm) in the skin, surrounded by areas of marked eosinophilic granulocyte infiltration, histiocyte proliferation, dermal fibrosis and compression atrophy of sebaceous glands; overlayered by either hyper- or hypokeratosis (Figure 3). The high number of cysts seen in tissue sections, the low Ct values in real-time PCR, and high PP values and titres in serological tests are indicative of high parasite load in chronically infected animals. In contrast to these results, half of the cattle diagnosed with *B. besnoiti* infection with the same methods in Switzerland had low ELISA PP values and IFAT titres [7]. The age of animals showing clinical signs in the present study (including three calves) was different from the recent outbreak of bovine besnoitiosis in Spain, where no disease was noted below one year of age [10].

This is the first report of autochthonous bovine besnoitiosis in Central-Eastern Europe, far away from the previously known endemic region (Figure 1). The outbreak of bovine besnoitiosis in eastern Hungary was evidently related to the import of subclinically infected French cattle into a region free from besnoitiosis, followed by local transmission, similarly to what was experienced in Germany, Italy and Switzerland [2,7].

### Conclusion

The current epidemiological situation in Europe warrants immediate action on both national and international levels to prevent further spread of besnoitiosis. Similar to diseases with comparable economic losses, cattle trading regulations concerning bovine besnoitiosis could be extended to include anamnesis records of donor herds, as well as prescribed serological testing and quarantine before cattle import from endemic regions.

### Ethical statement

The study was approved by the Ethics Committee of the involved Hungarian institutes, and was carried out with observing the national animal welfare regulations.

## Abbreviations

ELISA: Enzyme-linked immunosorbent assay; PP: Percentage positivity; IFAT: Indirect fluorescent antibody test; WB: Western blot; PCR: Polymerase chain reaction; Ct: Threshold cycle.

## Competing interests

No competing interests exist.

## Authors’ contributions

SH initiated and supervised the study, processed samples, extracted DNA and wrote the manuscript, AF reported and monitored the diseases, participated in sample collection, FB performed histopathological evaluation, RH arranged conditions of biopsy procedure and allocation of samples, WB performed serological and molecular analyses and contributed to the manuscript. All authors read and approved the final version of the manuscript.
